# Anatomical considerations for insertion of pedicular screw in cervicothoracic junction

**Published:** 2016-10-07

**Authors:** Morteza Faghih-Jouibari, Keisan Moazzeni, Amir Amini-Navai, Sara Hanaei, Sina Abdollahzadeh, Ramin Khanmohammadi

**Affiliations:** 1 Department of Neurosurgery, Shariati Hospital, Tehran University of Medical Sciences, Tehran, Iran; 2 Pasteur Hospital, Bam University of Medical Sciences, Bam, Iran

**Keywords:** Cervicothoracic Junction, Pedicle Screws, Dimension, Angulation

## Abstract

**Background:** This study aimed to investigate the pedicle dimension and angulation in cervicothoracic junction (CTJ) using the findings of computed tomographic (CT) to help accurate insertion of pedicular screw.

**Methods:** Forty three patients with high quality CT images of CTJ were evaluated. Pedicle width (PW), pedicle height (PH), pedicle axis length (PAL), transverse angle (TA) and sagittal angle (SA) were measured bilaterally from C6 to T2.

**Results:** Mean PW was 5.3 mm at C6, 6.2 mm at C7, 8.1 mm at T1 and 6.5 mm at T2. Males had larger pedicles than females. PH was greater than PW in all vertebrae. SA was relatively constant and around 15 degrees to horizontal plane. There was high variability of vertebral characteristics especially in PAL and TA.

**Conclusion:** Small diameter screws must be used for pedicular fixation in CTJ. Because of high variability of pedicle morphometry, CT scan is recommended in all patients before instrumentation.

## Introduction

Vertebral fixation in cervicothoracic junction (CTJ) is an essential part of treatment in different situations such as trauma, neoplasm, infection and degenerative diseases. Posterior instrumentation systems can provide greater biomechanical stability than anterior constructs in this region. In most cervical vertebrae, using lateral mass screw is the conventional method for posterior fixation but in lower cervical vertebra, lateral masses are small and pedicular screws may be required. However, pedicles of C6 and C7 are small and screw placement desires proper anatomical considerations. Besides, most surgeons use pedicular screw for posterior fixation of T1 and T2 but their pedicles have unique morphology that makes screw placement challenging by conventional techniques.

Different anatomy and relative infrequency with which the CTJ is involved in disease processes makes it a difficult area for spine surgeons to navigate. So, anatomical study of this particular region is of paramount importance to avoid or minimize neural and vascular complications. In this study, we investigated the pedicle dimension and angulation in C6 to T2 vertebrae based on computed tomographic findings to help accurate and safe cannulation of the pedicles.

## Materials and Methods

**Figure 1:**
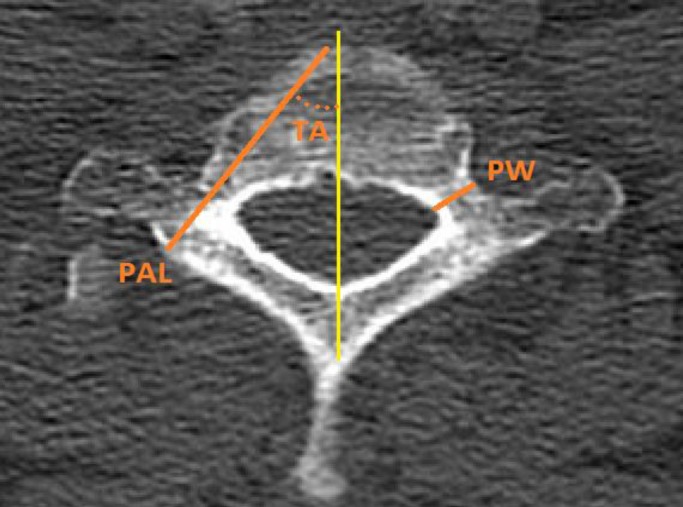
Illustrated method used to measure parameters in axial image

PW: Pedicle width; PAL: Pedicle axis length; TA: Transverse angle

Forty three patients who had cervicothoracic spinal multiplanar computed tomography (CT) imaging from August 2012 to December 2014 were evaluated. There were 22 males and 21 females who ranged in age from 22 to 60 years (mean, 38 years). We excluded patients with conditions potentially causing abnormal anatomy, such as previous spine surgery, neoplasm, fracture or spinal dysraphism. Axial CT images were attained with 1-mm slice thickness (Figure 1). Reconstruction into sagittal and coronal planes was then performed to measure various parameters (Figure 1); these parameters were measured bilaterally from C6 to T2.

      1- Pedicle width (PW): the narrowest outer cortical dimension of the pedicle in an axial plane

      2- Pedicle height (PH): superior-inferior diameter of the pedicle isthmus on the sagittal image 

      3- Pedicle axis length (PAL): the length from the laminar cortex through the center of the pedicle to the anterior wall of the vertebral body; this measurement provides an estimation of the potential screw length.

      4- Transverse angle (TA): the angle between PAL and a vertical line from the center of the vertebral body through the center of the spinous process (midline axis)

      5- Sagittal angle (SA): the angle between superior endplate and horizontal line in standing lateral cervicothoracic X-ray

Totally, 344 pedicles were measured. Continuous variables were expressed as mean ± standard deviations. Differences of variables were analyzed using t test. Statistical analyses were carried out by the SAS statistical analysis software package (version 9.1, SAS for Windows; SAS Institute, Cary, NC, USA).

## Results

Mean and standard deviation of PW, PH, PAL, TA and SA are shown in table 1. Mean PW and PH were not different significantly in left or right side (P = 0.31). Pedicular height was higher than PW in all vertebrae (P < 0.05).

Mean PW of male patients was 5.3 mm at C6, 6.4 mm at C7, 8.2 mm at T1 and 6.7 mm at T2. Mean PW in female patients was 5.2 mm at C6, 6.0 mm at C7, 8.1 mm at T1 and 6.3 mm at T2. Average PH in the males was 6.9 mm at C6, 7.5 mm at C7, 9.5 mm at T1 and 10.6 mm at T2. In the females, it was 6.8 mm at C6, 7.4 mm at C7, 9.0 mm at T1 and 10.4 mm at T2. Mean PW and PH were larger in males than in females in all four levels which were significant in C7 and T2 for PW and in T1 for PH (P < 0.05).

## Discussion

Anatomically, the CTJ has varying definitions. We define the CTJ as the superior end plate of the C6 vertebral body to inferior endplate of T2. The lowest two cervical vertebrae especially C7 have small lateral masses and pedicular screws may be required for fixation in many cases.

**Table 1:** Measurements of pedicular width (PW), pedicular height (PH), pedicular axis length (PAL), transverse angle (TA) and sagittal angle (SA)

**Vertebrae**	**PW (mm)** Mean ± SD	**PH (mm)** Mean ± SD	**PAL (mm)** Mean ± SD	**TA (degree)** Mean ± SD	**SA (degree)** Mean ± SD
C6	5.3 ± 0.9	6.8 ± 0.9	35.0 ± 3.7	42.0 ± 11.0	15.0 ± 2.1
C7	6.2 ± 1.1	7.5 ± 1.3	36.0 ± 4.6	38.0 ± 11.0	17.0 ± 2.1
T1	8.1 ± 1.4	9.2 ± 1.0	37.0 ± 4.3	35.0 ± 7.3	16.0 ± 2.8
T2	6.5 ± 1.0	10.5 ± 1.6	38.0 ± 4.3	22.0 ± 7.2	15.0 ± 3.0

SD: Standard deviation; PW: Pedicle width; PAL: Pedicle axis length; TA: Transverse angle; PH: Pedicular height; SA: Sagittal angle

The first and second thoracic vertebrae have small bodies and their pedicles have more medial trajectory than other thoracic vertebrae; thus conventional methods of pedicular screw insertion in thoracic vertebrae cannot be applied for these two vertebrae.^        1 ^ Investigating anatomic parameters of cervicothoracic vertebrae is necessary to avoid misplacement of pedicular screw and neurovascular injuries.^        2 ^

There are numerous publications studying dimensions of cervical and thoracic pedicles. Chazono et al. in their review of published data on cervical pedicle dimension did not find significant ethnic disparity.^   3 ^ The mean width of C6 to T1 pedicles in our study was comparable to other studies. Mean PW increased from C6 to T1 and then, decreased in T2. PW in C6 to T1 vertebrae are relatively small and assuming that screw diameter around two thirds of PW, their fixation desires smallest screws available with diameter of 3.5 to 4 millimeters. Otherwise, relatively large screws may result in pedicle wall violation which has been mentioned in many studies.^4^^-^^7^ 

Mean PH increased progressively from C6 to T2. PH was more than width in all of these vertebrae which shows ovoid shape of pedicle cross-section and underscores that mediolateral diameter of pedicle is more concerning during screw placement than superior-inferior diameter.

We observed that the mean PW and PH were larger in males than in females. This finding is similar to other studies.^8^^,^^9^ The intersex differences in PW and PH indicate that female patients should be given careful attention when considering pedicular fixation.

In addition to pedicular width, the proper angulation of the screw in the axial and sagittal planes is crucial in successful and safe cannulation of the pedicles. SAs of superior endplates in C6 to T2 vertebrae which marks SA of pedicular screws were relatively similar and mild caudal inclination (around 15 degrees) of screw seems appropriate. Transverse angulation of pedicles gradually decreased from C6 to T2 but they often had more medial angulation comparing to other thoracic vertebrae, so usual methods of screw insertion in thoracic spine are not ideal for CTJ.

In parallel to other studies,^   10 ^ we found high variability of vertebral characteristics especially pedicular medial angulation and PAL. Therefore, we recommend preoperative CT scan for candidates of instrumentation in CTJ.

## Conclusion

Pedicles in CTJ have small width and their fixation desires screws with diameter of 3.5 to 4 mm. Males have larger pedicles than females. Pedicles in CTJ have considerable variation of dimension and angulation; so, CT scan is highly recommended before instrumentation.

## Notes:

**How to cite this article:** Faghih-Jouibari M, Moazzeni K, Amini-Navai A, Hanaei S, Abdollahzadeh S, Khanmohammadi R. Anatomical considerations for insertion of pedicular screw in cervicothoracic junction. Iran J Neurol 2016; 15(4): 228-31.
